# Advancing adolescent health promotion in the digital era

**DOI:** 10.1093/heapro/daae172

**Published:** 2025-03-05

**Authors:** Rebecca Raeside

**Affiliations:** Susan Wakil School of Nursing and Midwifery, Faculty of Medicine and Health, The University of Sydney, Level 8, Susan Wakil Health Building, Western Ave, Camperdown, NSW 2006, Australia

**Keywords:** adolescents, digital health, health promotion, determinants of health, youth engagement, risk factors

## Abstract

Adolescents globally are calling for high-quality digital services to support and improve their health and well-being. Digital technologies are playing an increasing role in healthcare and whilst today's adolescents have been exposed to digital media since birth, there are unique challenges to their use that must be considered. This review aims to synthesize the literature on adolescent health promotion in the digital era. It provides evidence from adolescent perspectives and identifies that community-based and ‘digital only’ settings hold scope for further research to advance the field. The article recommends that when working with adolescents to develop digital health promotion tools, we should look to use youth engagement frameworks that are relevant to their context. Secondly, it demands stronger governance over digital media to protect adolescents, whilst allowing safe digital access. Finally, it demonstrates how listening to adolescents may help to address the emerging digital determinants of health and avoid exacerbating health disparities. Adolescents are powerful advocates to make global change. Stakeholders across research, policy and practice should examine how they incorporate adolescent voices to drive change in health promotion in the digital era.

**Contribution to Health Promotion**

- Today’s generation of adolescents are the largest generation in history and are calling for high-quality digital health and well-being services.
- Digital technologies may enable individuals to improve their health. However, it is essential to avoid exacerbating health disparities.
- This review synthesizes literature on adolescent health promotion in the digital era, incorporating the current evidence within various settings, adolescents perspectives towards digital health promotion and the impact of the digital determinants of health.
- Three key recommendations are provided: use of relevant youth engagement frameworks, stronger governance of digital media and addressing the digital determinants of health.

There are 1.8 billion adolescents globally, which is more than any other time in history (Partnership for Maternal, Newborn and Child Health [PMNCH], 2023). Emerging definitions of adolescence consider those between 10 and 24 years old, which corresponds more closely with both adolescent growth and the shift in social roles during this life stage (Sawyer *et al*., 2018). The adolescents of today are powerful advocates and leaders within their communities, with the potential to make substantial changes to our world. The Agenda for Action for Adolescents, led by the PMNCH and co-developed with adolescents highlights seven priorities for urgent action (PMNCH, 2023), one of which is to provide affordable, high-quality adolescent health and well-being services including digital services. To address this priority, we must examine the current state of adolescent health promotion in the digital era, and identify key areas for action within research, policy and practice.

## DEVELOPMENTS IN DIGITAL HEALTH PROMOTION

Digital technologies have become entwined in the lives of people globally, including playing an increasing role in their healthcare (Miller and West, 2009). This comes with the opportunity to use digital technologies to provide access to information and services, which were previously non-existent—including in health promotion (Nutbeam *et al*., 2021). Digital health promotion is defined as the applications of digital technologies to enable people to increase control over, and to improve, their health (Koh *et al*., 2021; World Health Organization, 2024). Improving technologies, greater connectivity and adoption of smartphones and wearable devices over time provide further potential for using digital technologies for health promotion (Koh *et al*., 2021). Not all countries globally have adequate healthcare coverage to provide access to health promotion information (World Health Organization, 2023), and digital technologies can be used to extend coverage and provide access. In addition, the COVID-19 pandemic accelerated the integration of digital health services into usual care (Mosnaim *et al*., 2020; Anthony, 2021; Dettori and Castiglia, 2022) and changed the landscape of healthcare. Digital health is here to stay, and it is vital to identify ways we can use technology to enable individuals to increase control over, and improve, their health.

However, many digital health initiatives focus only on individual responsibility for improving health behaviours, ignoring upstream contributors that contribute to health inequalities (Williams and Fullagar, 2019). It is recognized that the ability to improve health behaviours will be more impactful in a system that also supports them, requiring multi-sector collaboration to work towards a healthy and equitable future (Sims and Aboelata, 2019). Previous work has suggested that the digital domain is a ‘super’ social determinant of health (Hanebutt, 2023), as the gap in digital literacy and access between historically marginalized groups and others compounds existing health disparities. However, more recent literature discusses how the design, implementation and use of technology interact with the social determinants of health to influence health outcomes—such interactions are defined as the digital determinants of health (Chidambaram *et al*., 2024). The digital determinants of health refers to the technological factors that are incorporated to provide affordable, accessible and quality care to consumers enhancing their healthcare engagement and experience (Chidambaram *et al*., 2024), considering both the adoption of digital tools and health equity. It is vital that these determinants are considered to achieve health promotion through digital technologies.

## ADOLESCENTS AND DIGITAL HEALTH

Adolescence, 10–24 years, is a life stage of biological, emotional and social development (Laski, 2015). It is widely recognized that specific attention should be given to adolescent health, separate from children or adults (World Health Organization, 2014). Adolescence is often regarded as the ‘second window for opportunity’ (UNICEF Office of Research – Innocenti, 2017), where influence on brain development can be achieved through the experiences and environments to which they are exposed. Therefore, an opportunity exists to develop interventions that aim to improve adolescent health and well-being. Health behaviours including diet, physical activity, tobacco and alcohol use, often begin within adolescence. These established health behaviours often continue into adulthood to influence morbidity and mortality throughout life (Viner and Macfarlane, 2005). In the last 20 years, there have been limited changes to the way preventive health care has been delivered to adolescents (Schor, 2024). However, there are emerging challenges that adolescents face to maintaining healthy behaviours, including climate change, digital technologies, social media and the commercial determinants of health (The Lancet Public, 2024). Though adolescence is a window for opportunity, it is also a period of vulnerability due to neural plasticity, which may have lasting effects on adolescent development (DAHL, 2004). In particular, the commercial determinants of health, defined as ‘the systems, practices and pathways through which commercial actors drive health and equity’ (Gilmore *et al*., 2023), have a substantial impact on adolescent health and well-being (Pitt *et al*., 2024). Commercial actors are using traditional and contemporary marketing strategies to promote harmful products to adolescents (Montgomery and Chester, 2009; Soraghan *et al*., 2023). Given that adolescence is a period of both opportunity and vulnerability, there is a specific need for health promotion during adolescence in acceptable and engaging formats.

Today's adolescents are digital natives, and their lives are intertwined with technology from birth (Lusk, 2010). Though there are many benefits of digital media use (learning, exposure to new knowledge, and increased social support), there are also risks including negative effects on attention, exposure to misinformation and risks to privacy and confidentiality (Reid Chassiakos *et al*., 2016). Adolescents are typically the highest users of digital media, though access can vary in different parts of the world (Holly *et al*., 2023), and they use digital technologies in various ways to obtain health information. Research shows that they have been doing this for more than 20 years. A study by Skinner and colleagues in 2003 found that adolescents were using the internet to seek health-related information and acknowledged that this brings novel challenges for health professionals (Skinner *et al*., 2003). Over time, the ways adolescents seek health information have evolved. In 2010, Ito and Brown found that newer forms of digital media (e.g. social media and smart phones) were more attractive to adolescents for health information since they are more interactive and mobile (Ito and Brown, 2010). More recent studies have shown that adolescents are changing their behaviour based on information found online (Blázquez Barba *et al*., 2018; Raeside *et al*., 2022), yet the content that they are viewing has limited objectivity and transparency (Armstrong *et al*., 2021). Given that adolescents are digital natives and are seeking health information online, there are opportunities present to leverage digital technologies for health promotion among this age group.

Studies have been conducted to elucidate the effectiveness of digital interventions on improving health behaviours among adolescents. Rose and colleagues demonstrated that digital interventions to improve physical activity and diet among adolescents were effective in causing behaviour change, yet longer follow-ups were needed to determine whether behaviour change was sustained (Rose *et al*., 2017). Another systematic review confirmed these findings among web-based interventions for health behaviour change among adolescents (de Sousa *et al*., 2022). As the variety of digital settings grows, there is a need to evaluate emerging digital platforms. In addition, there has also been promising research demonstrating the benefits of digital interventions for mental health promotion among adolescents (Wright *et al*., 2023), and that digital interventions targeting preventive health behaviours have small yet positive effects on mental health and well-being outcomes (Raeside *et al*., 2023; Smout *et al*., 2024). Therefore, digital settings show a great deal of promise in delivering holistic health promotion information to adolescents. Yet, there are challenges that must be considered.

The following article aims to synthesize the literature on adolescent health promotion in the digital era. Four main areas are considered: (i) current settings for adolescent digital health promotion, (ii) adolescents perspectives towards digital health promotion, (iii) the relationship between the digital determinants of health and adolescent digital health promotion and (iv) areas for action in research, policy and practice.

## CURRENT SETTINGS FOR ADOLESCENT DIGITAL HEALTH PROMOTION

Digital health promotion for adolescents can occur across settings including ‘digital only’, school-based, primary care and community-based. A review by Stark and colleagues examining digital health promotion in different settings found that it is most commonly occurring in schools, communities and ‘digital only’ settings (Stark *et al*., 2022). However, most of the research has been conducted in high-income countries. A recent review of digital health promotion for children and adolescents found that 82% of the included studies were conducted in high-income countries, and a larger focus is needed on research and implementation of digital health promotion for adolescents in low- and middle-income countries (LMICs) (Oh *et al*., 2022), so that it is not only coming from a Western lens. Ferretti and colleagues explored some of the gaps and provided several important factors that must be addressed in LMICs including increasing digital access and literacy, involving adolescents in co-design, ensuring safety and monitoring of digital tools and improving technology governance (Ferretti *et al*., 2023b). ‘Digital only’ settings (e.g. mobile devices, web-based programs, social media, apps, telemonitoring devices) hold an important role in health promotion to adolescents as they can be delivered at scale. Though all examples described below are delivered in digital settings, some are also based within a setting that is physical. Below, we discuss the current evidence for digital health promotion in various settings.

School-based settings are important for digital health promotion amongst adolescents, given that they can reach large populations and have potential to be scaled to meet the needs of adolescents. In fact, most research on digital health promotion has occurred in schools (Oh *et al*., 2022). However, it must be considered whether this is an effective setting for digital health promotion. Digital health promotion programs may be used within the school environment when technology is available for use (e.g. Wi-Fi), yet adolescents may not be willing to use their own mobile data for access if no connectivity is available (Kennedy *et al*., 2018). A review by Champion and colleagues showed promise for school-based e-health interventions to improve lifestyle health behaviours including physical activity, fruit and vegetable intake and screen time. Yet longer follow-ups were needed to determine whether the behaviour change was sustained beyond the intervention period (Champion *et al*., 2019). In addition, important conditions have been identified to ensure effective implementation of digital health promotion programs in schools. These include that the program becomes a tool of choice in the school and links to existing school programs, that resources are invested to ensure uptake, that it is user-friendly, and that the platform engages participation of all (Dagenais *et al*., 2022).

Primary care—though not identified as one of the most common settings for digital health promotion—should be considered. In 2000, no studies on physical activity and nutrition interventions for adolescents within primary care were identified, digital or otherwise (Sallis *et al*., 2000). Studies among adults had previously shown promise; therefore, this study concluded that further research to demonstrate effectiveness among adolescents was warranted. Twenty years on, considerable research has occurred demonstrating promise for adolescent digital health promotion within primary care. Yet unique challenges of technology are highlighted including the disconnect between digital tools and clinical care, privacy and security concerns of adolescents and the value of digital health tools (Wong *et al*., 2020). In addition, ongoing issues with access to primary care services exist for adolescents (Sanci *et al*., 2005; Kang, 2018), especially those from minority groups (e.g. Indigenous populations) (Harfield *et al*., 2024). Adolescents desire to use technology with their health providers to have questions answered outside of visits, have greater access to providers as a method of building rapport, and for sharing data regarding their health behaviours between visits (Radovic *et al*., 2018). Effective policies and service planning are needed in collaboration with adolescents to ensure that digital solutions can be incorporated to deliver digital health promotion within primary care.

Community-based has many meanings with community acting as a setting, target, agent or resource (McLeroy *et al*., 2003). For this review, we focus on the community as a setting. Community-based approaches are important in digital health promotion as they can enable access to groups that may be particularly difficult to reach, for example, those who are socially disadvantaged or marginalized (Koh *et al*., 2021). There is limited research specific to adolescents in this setting. Digital formats are found to be appropriate for community health promotion when anonymity and flexibility are considered (Schroeer *et al*., 2021). For adolescents, this may be particularly beneficial for community-based peer support health promotion programs. Rose-Clarke and colleagues demonstrated that community-based, peer-facilitated interventions in LMICs are promising for improving adolescent health outcomes (e.g. improving mental health and reducing substance use and violence) (Rose-Clarke *et al*., 2019). No similar studies could be found in high-income countries. Hence, there is scope to focus research efforts on community-based digital health promotion programs. Examples of interventions in school-based, primary care, community and ‘digital only’ settings are described below in Table 1.

**Table 1:** Examples of digital health promotion interventions for adolescents in various settings

Setting	Description
School-based^a^	Country: 5 European countries (Austria, Germany, Greece, Sweden, and Belgium). Intervention: Healthy Lifestyle in Europe by Nutrition in Adolescence (HELENA). Computer-tailored program consisting of: (a) an introduction page; (b) a diagnostic tool; and (c) advice. Students received tailored feedback about their attitudes, self-efficacy, social support, knowledge, perceived benefits, and barriers related to their physical activity in the final part of the advice. Setting role: Intervention is delivered during school hours and guided by teachers. Duration: 1 month Ages: 12–17 years old.
Primary care^b^	Country: USA. Intervention: Electronic health risk behaviour screening with integrated feedback. Setting role: Intervention is delivered during primary care visit. Duration: 6 months. Ages: 13–18 years old.
Community-based^c^	Country: USA. Intervention: Text-messaging intervention to improve fruit and vegetable and healthy beverage intake in rural adolescents. Setting role: Intervention is delivered to rural-dwelling adolescents only. Duration: 8 weeks. Ages: High school students.
‘Digital only’^d^	Country: Australia. Intervention: Text message intervention to improve body mass index and lifestyle outcomes in adolescents who are above a healthy weight. Setting role: Intervention is delivered via digital setting with no restrictions on location (other than within one country). Duration: 6 months. Ages: 13–18 years old.

^a^ De Bourdeaudhuij *et al.* (2010).

^b^ Richardson *et al.* (2021).

^c^ Gustafson *et al.* (2019).

^d^Partridge *et al.* (2020).

## ADOLESCENTS’ PERSPECTIVES TOWARDS DIGITAL HEALTH PROMOTION

While it is well known that adolescents frequently use digital media, it is crucial to understand their acceptance of digital platforms for receiving health promotion information. Studies have been conducted in high-income countries to understand how adolescents are using technology to support their health. A study conducted in the USA found that adolescents are using technology to gather information, share their experiences, view others experiences and track behaviours and health goals (Radovic *et al*., 2018). An Australian study found that 78% of adolescents were using websites and 77% were using social media for seeking health promotion information. However, most participants found these only somewhat helpful (Armstrong *et al*., 2021). Adolescents also report using websites and social media differently for accessing health promotion information. For websites, they actively search for information, whereas on social media, they passively receive information presented to them (Raeside *et al*., 2022). Adolescents also desired health promotion information to be well-presented, credible and relevant to them (Raeside *et al*., 2022). Another study, which asked participants about social media specifically, had similar findings where adolescents desired information that was reliable, attractive and tailored to meet their needs (Plaisime *et al*., 2020). Together, findings demonstrate that adolescents are using digital media to obtain health promotion information and that they have specific requirements that must addressed for such information to be acceptable to them.

Ferretti and colleagues were the first to synthesize all studies that gather youth perspectives on digital health promotion (Ferretti *et al*., 2023a). Adolescents described features, which appealed to them including the quality of the user interface, a supplement to their personal efforts to maintain good health, informative and tailored content, sense of community, effective behaviour change and privacy and confidentiality (Ferretti *et al*., 2023a). Drawbacks to the use of digital health promotion tools were also raised, including friction with user experience, a lack of personalisation, privacy risks, insufficient human interaction, the risk for misinformation and poor evidence of effectiveness (Ferretti *et al*., 2023a). These findings can also be seen in a systematic review assessing effective design features for youth engagement in games for health promotion, where Schwarz and colleagues found that adaptability to suit the user’s needs, along with high-end graphics, and characters, which the user can identify with, were associated with higher user engagement (Schwarz *et al*., 2020). Adolescents have also reported that they want governments and technology companies to provide stronger regulation of online content and services that can protect them from both harm and misinformation, as well as increasing access to quality and trustworthy health information (Governing Healthy Futures Commission, 2021).

Significant investment is needed for the development of evidence-based digital health promotion interventions that are both relevant and appealing to adolescents and do not widen existing health disparities, which can be achieved through genuine youth engagement. A recent umbrella review found 99 articles describing the positive impacts of adolescent involvement in health research, which has benefits to adolescents themselves (increased knowledge and skills and personal development) and to the research (improved recruitment, data collection and analysis). Yet the quality of the evidence remains weak due to lack of reporting and evaluation (Warraitch *et al.*, 2024b). Challenges to involving adolescents in health research have also been identified (Warraitch *et al*., 2024a), many of which may be addressed by the use of youth engagement theories, guidelines or frameworks to guide adolescent involvement. Youth engagement theories, guidelines and frameworks have been used widely within the literature (Sanchez *et al*., 2024; Warraitch *et al.*, 2024c), which may be classified as power, process, impact or equity-focused. Examples of each focused framework are available in Table 2. Though there are many available, they are often narrow in scope, limited to one context and may depend on available resources (Sanchez *et al*., 2024).

**Table 2:** Examples of youth engagement theories, guidelines and frameworks

Focus	Description	Example
Power-focused	Focus is on identifying, describing or explaining who has leadership or power in partnership between youth and researchers.	Authentic Youth Participation^a^
Process-focused	Focus is on describing barriers, facilitators and other factors affecting youth engagement in research.	Youth Agency for Social Change Model^b^
Impact-focused	Focus is on describing and conceptualizing the potential impacts of youth engagement in research.	EIPARS Model^c^
Equity-focused	Focus is on identifying and proposing alternative approaches to conventional methods in research.	YPAR 2.0 Model of Research Engagement^d^

^a^ Hinkle *et al.* (2018).

^b^ Suleiman *et al.* (2006).

^c^ Norman and Skinner (2007).

^d^ Akom *et al.* (2016).

## RELATIONSHIP BETWEEN THE DIGITAL DETERMINANTS OF HEALTH AND ADOLESCENT DIGITAL HEALTH PROMOTION

Though digital health promotion interventions hold promise to improve adolescent health outcomes and improve access, they also have the potential to exacerbate existing health disparities. Digital health promotion interventions typically support individuals to improve health outcomes through behaviour change (Webb *et al*., 2010; Lustria *et al*., 2013). These often require individuals to have digital technology access and high levels of digital health literacy (Busse *et al*., 2022). As a result, evidence suggests that digital health interventions may be less effective for historically marginalized groups (Brewer *et al*., 2020). This is known as the digital divide, where disparities exist between those with digital access and digital literacy, and those without (Hanebutt, 2023). Among adolescent digital health promotion interventions which are growing in popularity, most do not consider factors that affect access and engagement (Rose *et al*., 2017; Raeside *et al*., 2023) and of those that do, few sought to implement strategies that would improve these when identified (Whitehead *et al*., 2024).

**Fig. 1:**
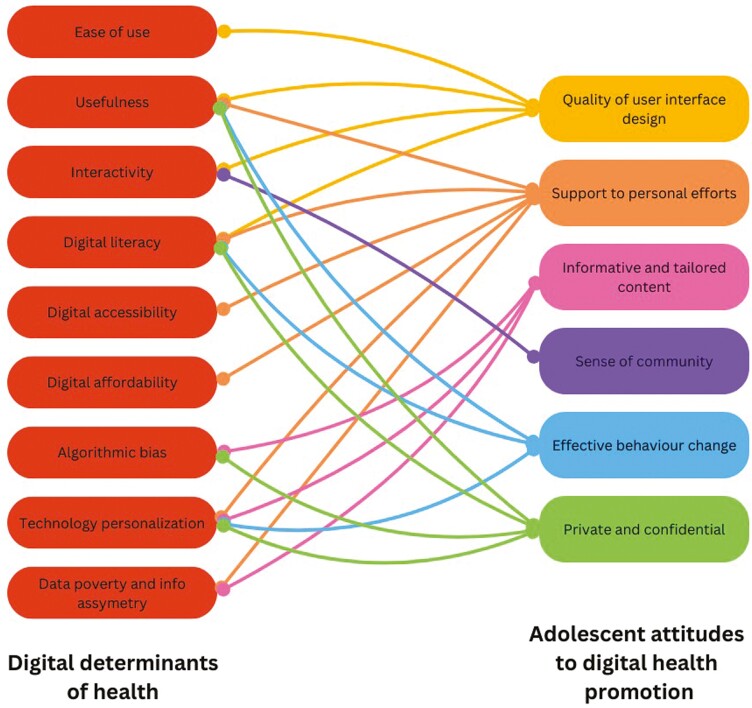
Concept map of digital determinants of health linked with adolescent attitudes to digital health promotion.

We can look to adolescent digital health promotion to drive change in addressing the digital determinants of health (Kickbusch and Holly, 2023). Many of the adolescent attitudes towards digital health promotion identified by Ferretti *et al*. (Ferretti *et al*., 2023a) are cross-cutting with dimensions of the digital determinants of health (Chidambaram *et al*., 2024). For example, technology personalization is one of the digital determinants of health, also cited by adolescents to be highly appealing. Furthermore, adolescents have called for high-quality, usable digital tools for health promotion, addressing further dimensions of the digital determinants of health including ease of use, usefulness, interactivity and digital literacy. Figure 1 depicts a concept map demonstrating cross-cutting themes between adolescent attitudes towards digital health promotion and the digital determinants of health. By genuinely engaging adolescents in development of digital health promotion interventions, we can seek to create digital health promotion tools, which do not widen existing health disparities.

Development of effective and equitable digital health promotion tools for adolescents cannot be achieved by youth engagement alone. Stronger governance and regulation around digital media is also crucial for addressing the digital determinants of health (Governing Healthy Futures Commission, 2021) and protecting the well-being of adolescents (Holly *et al*., 2023). Adolescents remain vulnerable as they are creating online identities whilst they continue to grow and develop offline (Israni *et al*., 2020). In summary, adolescents have unique views on the use of digital tools for health promotion, which must be explored and adhered to when developing tools for digital health promotion, and governments and technology companies have a crucial role to play in protecting adolescents in the digital world.

## AREAS FOR ACTION IN RESEARCH, POLICY AND PRACTICE

Digital health promotion for adolescents has strong potential for effectiveness. Yet, the need for health promotion among adolescents challenges the capacity of traditional healthcare systems and school settings. Current evidence demonstrates that there is scope for adolescent digital health promotion interventions particularly within community-based and ‘digital only’ settings. However, developing and implementing effective digital health promotion interventions for adolescents requires new approaches that engage adolescents throughout the entire research process and in the development of policies to protect them in a digital world. Three key areas for action are suggested:

### Use of relevant youth engagement frameworks

It is important for researchers to select theories, guidelines or frameworks that are relevant to their context. However, it is difficult to provide specific examples for each context as relevance will depend on systemic factors including time, resources, systems and expertise within the team. Studies, which synthesize theories, guidelines and frameworks (Sanchez *et al*., 2024; Warraitch *et al.*, 2024c) are a good starting place for those working to engage youth. It is acknowledged that further work is needed to provide youth engagement theories, guidelines and frameworks that are comprehensive, developed with youth, and can be applied across multiple contexts. Selection and application of the most appropriate youth engagement frameworks (taking systemic factors into account) in development of digital health promotion interventions will provide valuable reporting and evaluation guidance for those in research, policy and practice. This in turn will lead to generation of rigorous, higher-quality studies that will help to drive policy action.

### Stronger governance of digital media

Action is needed to strengthen governance through proactive policies from technology companies along with government-led legislation that acts to protect adolescent health and well-being, yet also allows them access to continue to explore the digital world safely (Roman-Urrestarazu *et al*., 2022). Effective legislation from governments may include regulation on the quality of health information on social media platforms, and regulation on marketing of unhealthy products. Furthermore, greater commitment from governments and educators to improve digital health literacy skills of adolescents is needed. Adolescents themselves should also be given the right to contribute to the development of these policies (United Nations, 1990), as their unique perspectives and experiences are key to shaping a digital environment that is safe, equitable and inclusive for all.

### Addressing the digital determinants of health

To ensure that all digital determinants of health are addressed, it is vital to seek representation from a broad range of adolescents when engaging them to develop digital health promotion tools. Without fundamental change to address the digital domain as a ‘super’ social determinant of health, the digital divide will only continue to be exacerbated. Frameworks have been developed to support the development of digital health tools that are equitable, and in line with the digital determinants of health across individual, interpersonal, community and societal levels (Richardson *et al*., 2022). Such frameworks will support those who develop digital health tools within academia and practice to ensure that they do not widen the equity gap.

Today’s adolescents have the power as the largest generation in history to make substantial social and system changes to our world. The development of high-quality digital health promotion tools that empower adolescents to improve health behaviours is within reach. Researchers, policymakers and those in practice must look to engage adolescents to drive change. This in turn will assist in the development of digital health promotion tools that are equitable and accessible for all.

## Data Availability

No new data were generated or analysed in support of this research.
